# Hypercalcemia of Malignancy and Colorectal Cancer

**DOI:** 10.14740/wjon953w

**Published:** 2016-04-03

**Authors:** Rodolfo J. Galindo, Isabela Romao, Ageliki Valsamis, Stuart Weinerman, Yael Tobi Harris

**Affiliations:** a Icahn School of Medicine at Mount Sinai, Division of Endocrinology, Diabetes and Bone Diseases, Mount Sinai St. Luke’s Hospital, 1111 Amsterdam Ave, Babcock Building 10th Floor, Room 1020, New York, NY 10025, USA; b Hofstra North-Shore LIJ School of Medicine, Division of Endocrinology Diabetes and Metabolism, 865 Northern Boulevard, Suite 203, Great Neck, NY 11021, USA

**Keywords:** Hypercalcemia, Parathyroid hormone-related peptide, Calcitriol, Colorectal cancer, Combined mechanism of hypercalcemia

## Abstract

Our aim is to describe the association between colorectal cancer (CRC) and humoral hypercalcemia of malignancy (HHM). Causes of hypercalcemia of malignancy include parathyroid hormone-related peptide (PTHrP) secretion, local osteolysis, calcitriol production and ectopic parathyroid hormone (PTH) secretion. Hypercalcemia of malignancy in patients with CRCs is a rare scenario. A patient with anal squamous cell carcinoma was admitted with hypercalcemia, suppressed PTH and hypophosphatemia. He was found to have metastatic anal squamous cell carcinoma to the liver. Further evaluation revealed elevated PTHrP and 1,25-dihydroxyvitamin D and low 25-hydroxyvitamin D. Over a 5-month course, the hypercalcemia responded poorly to bisphosphonates, transiently to prednisone, but showed marked improvement with chemotherapy. A review of English language publications in Pubmed and a reference search of retrieved articles revealed 29 cases of CRC causing PTHrP-mediated hypercalcemia. Most patients were middle-aged men (mean ± SD: 56.7 ± 13.4 years), with advanced metastatic cancer (85% with hepatic metastasis) and severe hypercalcemia (mean ± SD: 15.6 ± 1.9 mg/dL, 62% with Ca > 14). This condition is associated with high mortality (79%) and short survival (median 54.5 days, CI: 21 - 168). Despite being uncommon, HHM (PTHrP-mediated) should be considered in patients with metastatic CRC presenting with hypercalcemia. Clinicians should be aware that combined etiologies may be present, particularly in cases of resistant hypercalcemia. Treatment of the underlying malignancy is essential for calcium control.

## Introduction

Malignancy is the most common cause of hypercalcemia in hospitalized patients [1]. The differential diagnosis of malignancy-associated hypercalcemia includes, in decreasing order of frequency [2, 3], humoral hypercalcemia of malignancy (HHM) secondary to secretion of parathyroid hormone-related peptide (PTHrP), generally by squamous cell tumors [4, 5]; local osteolytic hypercalcemia, caused by cytokines, chemokines and PTHrP [5, 6]; calcitriol-mediated hypercalcemia, seen most commonly in lymphomas and leukemias [5, 7]; and rarely ectopic hyperparathyroidism [5, 8]. These mechanisms are not mutually exclusive and combined causes are rare but should also be considered.

HHM is frequently seen in squamous cell cancers of the head and neck, esophagus, cervix and lung [3], as well as breast cancer [4], renal cell carcinoma [9] and hematological malignancies [5]. Additionally, Asa et al reported elevated expression of PTHrP in pheochromocytomas, thyroid carcinomas and small cell lung carcinomas [10].

Here we present a rare case of colorectal cancer (CRC) and hypercalcemia of malignancy with elevation of PTHrP and calcitriol. We also provide a literature review of HHM in CRC. To our knowledge, this is the first reported case of calcitriol-induced hypercalcemia in human CRC.

## Case Report

**Figure 1:**
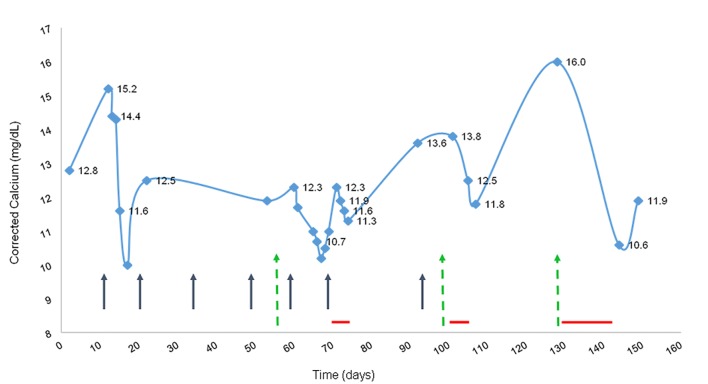
Corrected calcium and response to therapy. Black arrows represent treatment with zoledronic acid; green dashed-arrows represent chemotherapy and dexamethasone; red horizontal lines represent prednisone treatment (40 mg daily).

A 58-year-old man presented with syncope and falls during postural changes. He had been diagnosed with anal squamous cell carcinoma 1 year prior, and had responded well to chemotherapy. No systemic disease had been found on initial staging. He also had a history of bipolar disorder but was never treated with lithium. His medications included gabapentin and docusate. On presentation, he was bradycardic to 48 bpm and had orthostatic hypotension. Physical exam revealed temporal wasting, dry mucous membranes, leg edema and mild confusion. Laboratory evaluation showed hypercalcemia (corrected calcium of 15.3 mg/dL, reference range 8.4 - 10.5 mg/dL), suppressed parathyroid hormone (PTH) of 8.5 pg/mL (15 - 65 pg/mL) and hypophosphatemia (PO_4_ of 1.9 mg/dL, reference range 2.5 - 4.5 mg/dL). CT scans of the head and chest were unremarkable. Bone scan did not reveal any lesions. CT scan of the abdomen and pelvis demonstrated multiple solid hepatic lesions. A liver biopsy revealed metastatic squamous cell carcinoma. Further evaluation for hypercalcemia revealed elevated PTHrP (6.7 pmol/L, reference range < 2 pmol/L), decreased 25-hydroxyvitamin D (25(OH)D of 27.2 ng/ml, reference range 30 - 100 ng/mL) and increased calcitriol (75 pg/mL, reference range 18 - 64 pg/mL). A diagnostic evaluation for causes of elevated calcitriol was unrevealing. Fibroblast-growth-factor 23 and angiotensin-converting enzyme levels were within normal limits. Blood, urine and spinal fluid cultures revealed no growth of any organism, and extensive virology testing showed only active hepatitis C virus infection. Imaging studies did not identify granulomatous disease. The patient’s calcium level improved transiently with standard therapy with saline hydration, calcitonin and zoledronic acid, and he was discharged home. A few weeks later, the patient was again admitted and had elevated CCa, PTHrP and calcitriol (12.3 mg/dL, 11 pmol/L and 205 pg/mL, respectively), which no longer responded to zoledronic acid, despite several courses of therapy. He received chemotherapy - cisplatin, 5-fluorouracil, and dexamethasone - and his CCa levels transiently improved. After rapid recurrence of hypercalcemia, prednisone 40 mg daily was started and calcium levels began to decrease. The patient stopped taking his prednisone due to concern about elevated blood glucose and his calcium immediately increased again. Over a period of 5 months, the hypercalcemia responded weakly to bisphosphonates and transiently to prednisone but recurred immediately after cessation of therapy, as shown in Figure 1. Only chemotherapy achieved a significant improvement in calcium levels.

## Discussion

### Literature review

A Pubmed search of English language articles with multiple combinations of the terms including hypercalcemia, colorectal/anal cancer, metastasis, hypercalcemia of malignancy, PTHrP, calcitriol and combined causes, was performed. Related citations and cited references from retrieved articles were also reviewed. Fisher’s exact tests were performed to assess the relationship between calcium level and attributable death and between PTHrP level and attributable death. A survival distribution was estimated using the Kaplan-Meier product-limit method, whereby a survival time was considered “censored” at the time of last follow-up if the patient was alive or lost to follow-up. Comparisons of time-to-death for subjects with varying levels of calcium and PTHrP were each carried out using the log-rank test.

Our search revealed 29 cases of CRC with hypercalcemia due to PTHrP secretion (Table 1) [11-36], of which only five were anal squamous carcinoma [17, 24, 30, 32, 34]. Of the remaining cases, 18 described carcinoma of the colon and six of the rectum. The mean patient age was 56.7 years (mean ± SD: 56.7 ± 13.4 years), and 55% were male (in two cases, gender was not reported). Twenty-one cases involved metastatic disease, of which 85% had hepatic involvement. Total calcium levels ranged from 11.9 to 18.4 mg/dL (mean ± SD: 15.6 ± 1.9 mg/dL). Most patients (62%) presented with severe hypercalcemia (Ca > 14 mg/dL). Cases published prior to 1993 refer to a PTH-like substance, and the term PTHrP as a defined protein appears thereafter, as clinically useful assays to measure PTHrP became available [4]. PTHrP levels were reported in 14 cases, and ranged from 3.4 to 162 pmol/L (mean ± SD: 27.07 ± 40.2 pmol/L). The median overall time-to-attributable death was 54.5 days (95% CI: 21 - 168). Neither the relationship between calcium and death nor PTHrP and death was found to be statistically significant (P = 0.1142 and P = 1.0, respectively). Based on the log-rank test, there was no significant difference between those who had calcium level < 12, 12 - 14 or > 14 with respect to the time-to-attributable death (P < 0.2899). There was also no significant association between calcium level and PTHrP (P = 0.76).

**Table 1:** Cases of Colorectal Cancer and Humoral Hypercalcemia of Malignancy

Case number	Year (reference)	Age	Sex	Histological diagnosis	Metastasis	Calcium (mg/dL)	PTHrP (pmol/L)
1	1963 [35]	77	M	Cac	Li, LN	14	NR
2	1969 [36]	46	M	Cac	Li	15	NR
3	1969 [25]	79	M	Cc	NR	17.8	NR
4	1980 [18]	43	M	Cas	Li, P, K	18.4	NR
5	1980 [18]	39	M	Ras	Li, A	15.8	NR
6	1985 [26]	67	F	Ras*	Li, P, T, B, Per, Ad	16	NR
7	1987 [23]	41	F	Cas	Li	NR	NR
8	1987 [12]	41	F	Cas	Li, LN	18.4	NR
9	1989 [11]	74	M	Cas	Li, P	14.5	NR
10	1991 [22]	58	M	Rac	Li	17.6	NR
Cases reported after commercial assays became available							
11	1993 [16]	NR	NR	Cc	NR	NR	10
12	1993 [16]	NR	NR	Cc	NR	NR	20
13	1994 [19]	58	M	Cas	Li	11.9	25
14	1995 [14]	52	F	Rac	Li	18	162
15	1995 [24]	60	M	Ac	Pr, LN	17.3	33.2
16	1996 [31]	75	M	Cac*	Li, LN	13.2	36.7
17	1996 [27]	37	F	Cas	Li, LN	NR	NR
18	1998 [29]	47	M	Cc	NR	16	NR
19	1999 [20]	54	F	Cac*	Li, LN	15.2	18
20	1999 [20]	63	M	Rac	Li, P, LN, S	17	9.3
21	2001 [33]	76	F	Cas	Li, LN, S	13.6	25.7
22	2002 [21]	42	M	Cac*	Li, LN, B, S	16.2	12.1
23	2005 [15]	78	F	Cas	No	13.2	3.7
24	2005 [28]	54	F	Cac	Li, LN, P	14.2	13.5
25	2006 [13]	59	F	Rc*	LN	12.7	6.4
26	2008 [32]	57	M	Ascc	No	14	NR
27	2009 [17]	40	F	Ascc	No	15.8	HIGH
28	2010 [30]	52	F	Ascc	No	17.2	NR
29	2011 [34]	64	M	Ascc	LN	17.6	3.4

Ca: highest calcium (mg/dL); PTHrP: highest parathyroid hormone-related peptide (pmol/L). Histology: Cac: colon adenocarcinoma; Cas: colon adenosquamous carcinoma; Rc: rectal carcinoma; Rac: rectal adenocarcinoma; Ras: rectal adenosquamous; Cc: colon cancer unspecified; Ac: anal carcinoma; Ascc: anal squamous cell carcinoma. *Neuroendocrine features. Metastasis: Li: liver; LN: lymph node; P: pulmonary; Per: peritoneum; Ad: adrenal; B: bone; T: thyroid; NR: no reported; No: no metastasis; S: skin; Pr: prostate.

Our search did not reveal any case of calcitriol-induced hypercalcemia in patients with CRC, making this the first reported case. Moreover, we found only five cases of hypercalcemia from combined production of calcitriol and PTHrP: three ovarian adenocarcinomas, one renal cell carcinoma, and one pancreatic cancer with neuroendocrine features (Table 2) [37-41]. In this group, patients had moderate to severe hypercalcemia (12.9 - 18.9 mg/dL) on presentation. PTHrP ranged from 3.4 to 60.4 pmol/L and calcitriol ranged from 71.5 to 285 mg/dL. Notably, in four of the five cases, the patients survived the hypercalcemic episode [37-39, 41].

**Table 2:** Cases of Combined Paraneoplastic Production of Parathyroid Hormone-Related Peptide (PTHrP) and Calcitriol

Case number	Year (reference)	Age	Sex	Histological diagnosis	Metastasis	Calcium (8.4 - 10.5 mg/dL)	PTHrP (< 2 pmol/L)	Calcitriol (18 - 64 pg/mL)
1	1991 [39]	70	F	Oac	No	13.08	NR	285
2	2000 [38]	54	F	Oac	No	16.8	10	104
3	2006 [37]	74	F	Oac	No	12.8	60.4	116
4	2007 [41]	59	M	PNE	No	18.9	7.3	71.5
5	2009 [40]	57	M	RCC	Li	12.9	3.4	79

RCC: renal cell carcinoma; Oac: ovarian adenocarcinoma; PNE: pancreatic neuroendocrine tumor; Ascc: anal squamous cell carcinoma. Metastasis: Li: liver. Calcium, PTHrP and calcitriol: highest reported values.

### Characteristics of HHM and CRC

We report a case of combined PTHrP and calcitriol-induced hypercalcemia from CRC, adding to the literature of patients with CRC with HHM. As was the case for our patient, HHM in CRC typically occurs in middle-aged patients with advanced metastatic disease, usually to the liver. This condition is associated with severe hypercalcemia (62%), high mortality (79.1%), and short survival (median: 54.5 days). We did not find a statistically significant relationship between the reported calcium levels and length of survival. However, calcium levels reported in these cases represent only one point in time and do not reflect response to treatment, overall burden of disease, or other factors that affect survival. In the cases with reported PTHrP values, we found no significant association between PTHrP and calcium. One explanation for these findings may be that the assay for PTHrP was newly discovered and may not have been standardized between laboratories. Additionally, as this study had a very small sample size, there may have not been sufficient power to detect a small, yet clinically meaningful, difference in survival among groups.

### Paraneoplastic PTHrP production and CRC

This patient with metastatic CRC and recurrent hypercalcemia had a unique etiological mechanism: combined paraneoplastic production of PTHrP and calcitriol. PTHrP is a hormone functionally and structurally related to PTH. In 1941, Albright was the first to suggest that a hypernephroma was secreting a PTH-like substance [35]. It was not until the 1980s - 1990s that it was biochemically characterized and named PTHrP by a series of studies [42]. PTHrP selectively binds and activates the PTH receptor-1 and produces similar effects to those of PTH. It causes hypercalcemia by increasing osteoclastic resorptive activity and renal calcium reabsorption and causes hypophosphatemia by decreasing phosphorus reabsorption [2, 42, 43]. PTHrP differs from PTH, however, in that it decreases osteoblastic activity leading to unrestricted bone resorption [43, 44] and it is associated with lower calcitriol levels due to weaker stimulation of 1-alpha hydroxylase [45]. PTHrP is frequently produced by squamous cell cancers of the head and neck, esophagus, cervix and lung [3], as well as breast cancer [4], renal cell carcinoma [9] and hematological malignancies [5]. Additionally, Asa et al reported elevated expression of PTHrP in pheochromocytomas, thyroid carcinomas and small cell lung carcinomas [10]. Our review revealed 29 additional cases from CRCs.

### Management of PTHrP-mediated hypercalcemia (HHM)

Management includes general treatment of hypercalcemia along with specific therapies to target the pathophysiological mechanism. Hypercalcemia induces free water diuresis that may lead to volume depletion. Moreover, patients with malignancies may have decreased oral intake further worsening their nutritional and volume status. Thus, saline hydration is imperative for most patients. Calciuresis with loop diuretics is only recommended in those cases with volume overload or after adequate volume repletion is achieved.

Agents that block PTHrP-mediated increased bone resorption, such as pamidronate and zoledronic acid, will target the underlying disorder. Zoledronic acid is considered first-line, as it is highly effective in patients with HHM, benefiting more patients, and leading to a more rapid decrease in calcium than pamidronate [46, 47]. Calcitonin may transiently lower calcium for 24 - 48 h. Denosumab, a human monoclonal antibody that inhibits the receptor activator of nuclear factor kappa-b ligand, was recently shown to decrease serum calcium in 64% of patients with HHM despite recent intravenous bisphosphonate treatment [48]. Vitamin D deficiency, very common in cancer patients [49], may predispose them to severe and prolonged hypocalcemia from anti-resorptive therapy, thus those with subclinical osteomalacia should start vitamin D supplementation [50]. Our patient initially responded to zoledronic acid, but subsequently became refractory to therapy despite having a PTHrP level thought to be optimally responsive to bisphosphonates [16].

### Calcitriol-mediated hypercalcemia

**Figure 2:**
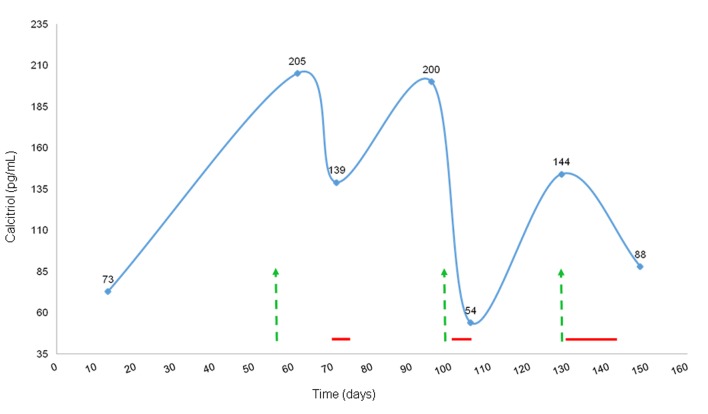
Calcitriol levels and response to steroids and chemotherapy. Green arrows represent chemotherapy and dexamethasone; red horizontal lines represent prednisone treatment (40 mg daily).

The minimal response to bisphosphonate therapy may have been due to the presence of a second mechanism of hypercalcemia: calcitriol-mediated hypercalcemia. Our patient had markedly elevated calcitriol levels on several measurements, as shown in Figure 2, with no other identifiable cause. Calcitriol (1,25-dihydroxyvitamin D), the active form of vitamin D, is converted from 25(OH)D by the renal 1-alpha hydroxylase, which is stimulated by PTH, and causes hypercalcemia by increasing intestinal calcium absorption. The 25-hydroxyvitamin D3 1-alpha-hydroxylase, encoded by the CYP27B1 gene, is also found outside of the kidney in cells including macrophages [51], but its normal role in these tissues is unclear [52]. Extra-renal over-expression of this gene is responsible for the paraneoplastic production of calcitriol seen in lymphomas and leukemias [53, 54]. Recently, a large retrospective series of 101 patients with calcitriol-mediated hypercalcemia by Donovan et al reported that sarcoidosis was the most common cause (49%), followed by hematological malignancies (17%) and granulomatous infections (8%). Solid tumors comprised only 5% of cases and included ovarian cystadenocarcinoma, seminoma, and metastatic squamous cell carcinoma of the tongue and non-small cell carcinoma of the lung. The median calcitriol level in patients with malignancy was 184 pmol/L (interquartile range 140 - 230 pmol/L) [7]. A review of rare causes of calcitriol-mediated hypercalcemia also reported dysgerminomas, leiomyoblastoma and squamous cell bronchogenic carcinoma [55]. Additionally, expression of 1-alpha hydroxylase has been demonstrated in malignant and normal colon cells, with higher mRNA expression in the less-differentiated tumors, compared to normal peri-tumoral cells [56], although hypercalcemia has not been reported.

Since the discovery of fibroblast growth factor-23 (FGF-23), new insights in the metabolism of phosphorus and 1,25-dihydroxyvitamin D have been proposed. FGF-23 is a bone-derived hormone that inhibits phosphorus reabsorption, resulting in hypophosphatemia. FGF-23 also inhibits 1-alpha hydroxylase and stimulates 24-hydroxylase resulting in decreased levels of calcitriol [57]. Our patient had low phosphorus levels, but the fact that the FGF-23 levels were within normal limits excluded any mechanistic implications.

### Management of calcitriol-induced hypercalcemia

As above, general measures, such as saline hydration, are indicated. Additionally, limiting sun exposure and calcium intake will target the underlying mechanism of hypercalcemia. Further treatment involves glucocorticoids, which inhibit extra-renal 1-alpha hydroxylase [54, 58-60], leading to a rapid decrease in calcitriol followed by a later decrease in serum and urinary calcium [61]. The anti-inflammatory/cytotoxic properties of glucocorticoids, leading to less 1-alpha hydroxylase activity, have been proposed as an additional mechanism; however, this effect was not seen in patients with lymphoma [54] and sarcoidosis-related hypercalcemia [61]. Typical prednisone doses range from 10 to 40 mg per day but cancer patients may require higher doses [54, 61].

### Combined production of PTHrP and calcitriol

Combined secretion of PTHrP and calcitriol by tumors is extremely rare and has only been reported in five cases: one renal cell carcinoma [40], another in a pancreatic neuroendocrine tumor [41], and three in ovarian cancers [37-39] (Table 2). We report the sixth case of combined production of PTHrP and calcitriol. In three of the previously reported cases, the hypercalcemia did not respond to bisphosphonates [39-41]. Steroids improved calcium levels in one patient who failed to respond to two doses of pamidronate. Hypercalcemia improved in two cases after surgery [37, 39]. In a third case, calcium normalized after the second surgery, and had transiently responded to bisphosphonates [38]. Ultimately, chemotherapy induced resolution of the hypercalcemia in most cases, emphasizing the importance of targeted therapy against the tumor.

Our patient had elevated levels of both PTHrP and calcitriol. We were unable to confirm elevated 1-alpha hydroxylase activity in the liver metastases and surrounding cells due to lack of tissue. It is therefore possible that the elevated calcitriol levels were due to stimulation of renal 1-alpha hydroxylase by the PTHrP, rather than extra-renal overproduction of 1-alpha hydroxylase, while PTH remained suppressed during the disease course. The effect of PTHrP on renal 1-alpha hydroxylase is somewhat controversial. PTHrP has been shown to cause lower levels of renal conversion of 25(OH)D to calcitriol than does PTH [43], and Schilling et al demonstrated that PTHrP does not influence calcitriol levels in patients with HHM [62]. Horwitz and colleagues used high doses of PTHrP [1-36] and PTH [1-34] infusions to demonstrate that PTHrP does have a stimulatory effect on renal calcitriol production, but much weaker than that of PTH [45, 63]. More recently, this same group demonstrated that PTHrP, at the lowest required dose to induce hypercalcemia, produced a decrease of calcitriol levels in healthy volunteers [64]. Thus, PTHrP seems not to increase calcitriol to levels similar to that of PTH. Given the weak effect of PTHrP, it is unlikely that the high levels of 1,25-dihydroxyvitamin D seen in our patient were due to its effects.

In our patient, bisphosphonate therapy did not consistently decrease calcium, while prednisone treatment significantly reduced calcitriol and decreased the calcium level. This finding was also reported by Shivnani et al, the only case of combined etiology with confirmed tumoral expression of 1-alpha hydroxylase [40]. Moreover, both calcitriol and calcium levels rose rapidly after the discontinuation of prednisone. The response to glucocorticoids as well as this patient’s markedly elevated calcitriol levels (> 200 pmol/L untreated) supported our hypothesis that he had both PTHrP and calcitriol-mediated hypercalcemia.

### Conclusions

We present a rare case of combined PTHrP and calcitriol production by a metastatic anal cancer. Overall, the prognosis is poor in patients with CRC and PTHrP-induced hypercalcemia. Bisphosphonate and glucocorticoid therapy can be temporizing in PTHrP-induced and calcitriol-induced hypercalcemia, respectively, but effective anti-cancer therapy is essential to the management of hypercalcemia of malignancy.

## Abbreviations

PTH: parathyroid hormone
PTHrP: parathyroid hormone-related peptide
25(OH)D: 25-hydroxyvitamin D
1,25(OH)_2_D: 1,25-dihydroxyvitamin D or calcitriol
HHM: humoral hypercalcemia of malignancy
CRC: colorectal cancer
